# Congenital Melanocytic Nevi With Meyerson Phenomenon: Two Case Reports and Review of the Literature

**DOI:** 10.5826/dpc.1003a64

**Published:** 2020-06-29

**Authors:** Ambra Di Altobrando, Iria Neri, Annalisa Patrizi, Michela Tabanelli, Cosimo Misciali, Carlotta Baraldi, Francesco Savoia

**Affiliations:** 1 Department of Experimental, Diagnostic and Specialty Medicine, Dermatology, University of Bologna, Italy; 2 Dermatology Unit, AUSL della Romagna, Ravenna, Italy

**Keywords:** congenital melanocytic nevus, Meyerson phenomenon, dermoscopy, dermatopathology, topical steroids

## Introduction

Meyerson phenomenon (MP) is an uncommon condition consisting of a symmetric area of eczema encircling a preexisting central cutaneous lesion such as a melanocytic nevus, a nonmelanocytic skin neoplasia, or a melanoma. MP has been reported to occur both in acquired melanocytic nevi in adults and congenital melanocytic nevi (CMNi) in the pediatric population [1].

## Case Presentations

### Case 1

**Figure 1:**
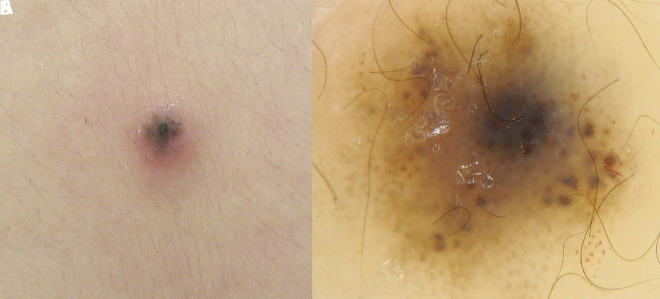
(A) A small congenital melanocytic nevus with Meyerson phenomenon. (B) Dermoscopy revealed a multicomponent pattern, with atypical globules and blotches of pigment, a central blue veil, along with perilesional erythema.

A 2.5-year-old boy was referred to us for a small CMN of the back encircled by an eczematous patch (Figure 1A). The patient, who had been suffering from atopic dermatitis since birth, complained of itching. Dermoscopy revealed a multicomponent pattern, with atypical globules and blotches of pigment, a central blue veil, along with perilesional erythema (Figure 1B). Therapy with a topical mometasone furoate led only to transient improvement, with recurrence after discontinuation and with persistence of the alarming dermoscopic features. Surgical excision of the lesion was performed and histology was suggestive of a CMN with MP. The excision of the lesion led to complete healing.

### Case 2

**Figure 2:**
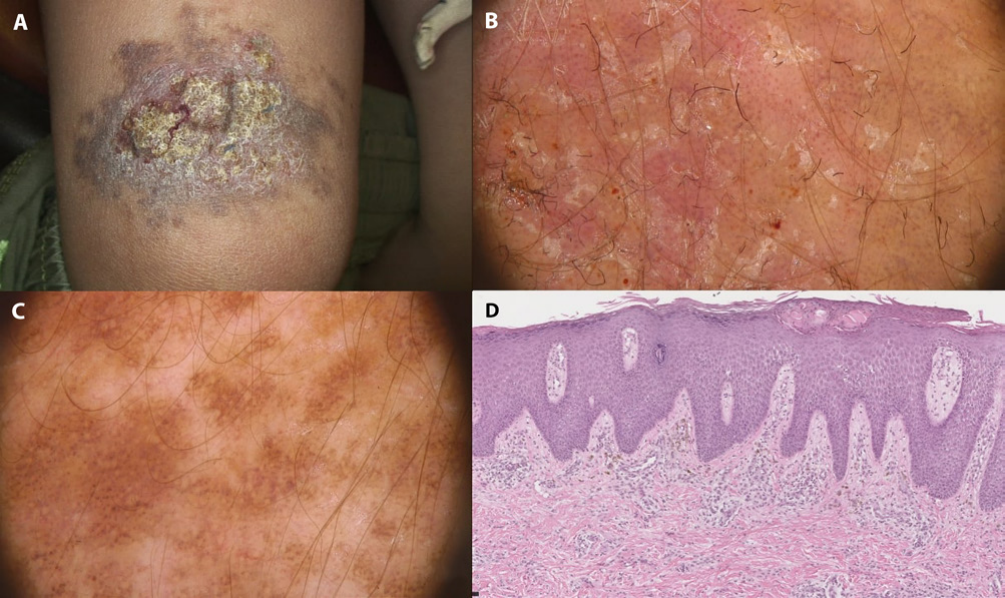
(A) A medium-sized congenital melanocytic nevus surmounted by yellow crusts and fissures. (B) Dermoscopy showed scales, glomerular/dotted vessels, and short linear black fibers stuck to the eczematous surface. (C) Areas with a homogeneous pigmentation were observed on dermoscopy. (D) Histopathology showed the features of a normal congenital melanocytic nevus associated with a chronic dermatitis.

A 6-year-old boy was referred to us for a medium-sized CMN of the right thigh that had progressively changed over the previous 12 months. On physical examination, a brown patch with ill-defined margins, 6 cm in maximum diameter, surmounted by yellow crusts and fissures, was observed (Figure 2A). The patient complained of itching and pain. He had suffered from atopic dermatitis during the first years of life, in remission at the time of consultation. Dermoscopic examination revealed areas characterized by pigment network and areas with a homogeneous pigmentation, in association with scales and glomerular and dotted vessels (Figure 2, B and C). Moreover, short, linear black fibers of clothing entrapped in the irregular eczematous surface of the lesion were detected. The use of clobetasol propionate 0.05% cream led to a reduction of the inflammation, without complete healing of the eczema, which recurred immediately after treatment discontinuation. A punch biopsy was performed and histopathology showed the features of a normal CMN associated with a chronic dermatitis, allowing the diagnosis of CMN with MP (Figure 2D).

Because of the severe pruritus and the chronic course despite therapy, seriate excisions were planned and performed, in order to achieve complete healing.

## Discussion

MP involving CMNi in the pediatric population is rare (Table 1). The most important differential diagnosis is melanoma, because the halo of eczema may cause clinical and worrisome dermoscopic features, including “blue-white structures” and “blue areas” [1].

Although MP can have a spontaneous complete resolution when associated with CMN, some authors have reported a chronic course with complete healing achieved only after excision of the CMN [2]. The use of potent topical steroids is associated with faster healing, even though recurrence of MP can occur within the same nevus or in different lesions [2].

## Conclusions

Our cases highlight the fact that in children MP in CMNi can have a chronic course, with severe eczema, itch, and pain, and can be responsible for alarming dermoscopic features, simulating a melanoma.

## Figures and Tables

**Table 1:** Cases of Congenital Melanocytic Nevi With Meyerson Phenomenon in the Pediatric Population

Ref.	No. of Patients	Age	Gender	Location	Type of Lesion	Atopic Dermatitis	Therapy	Therapeutic Response	Evolution
Richey [1]	1	4 years	F	Back	Congenital melanocytic nevus	No	—	—	—
Rolland [2]	5	Mean age 3.6 years (2 weeks-8 years)	3 M, 2 F	Upper extremity, ankle, trunk, arm and leg, thigh	Congenital melanocytic nevus	2 out of 5 patients	Topical steroids	Improvement in 3 cases, no improvement in 1 case; improvement with recurrence after suspension of treatment in 1 case, no treatment in 1 case	Decrease in pigmentation of nevi in 1 case, no follow-up in 1 case, no improvement over time in 1 case, 1 case lost at follow-up, improvement over time in 1 case
Weijns [3]	1	4 months	M	Upper leg	Congenital melanocytic nevus	—	—	—	—
Tauscher [4]	1	7 months	M	Arm	Congenital melanocytic nevus	—	—	—	—
Pižem [5]	57	Mean age 39 years (14–81)	53% F, 47% M	Back, abdomen, upper extremities, lower extremities, breast	16 acquired nevi, 3 congenital nevi, 2 Spitz nevi, 29 dysplastic nevi, 14 melanomas	15% of recalled patients reported a history of atopy	—	—	—
Our cases	2	2 years, 6 years	2 M	Back and thigh	2 congenital melanocytic nevi	Yes	Topical steroids; surgical excision	Improvement with topical steroids but recurrence after suspension of treatment	Complete healing of eczema with surgical excision
